# Comparative evaluation of rapid diagnostic test and peripheral smear microscopy for malaria diagnosis

**DOI:** 10.6026/973206300212795

**Published:** 2025-08-31

**Authors:** Mayuri Bhise, Garima Anandani, Parth Goswami, Abhishek Padhi, Ashwini Agarwal

**Affiliations:** 1 Department of Microbiology, All India Institute of Medical Sciences, Rajkot, Gujarat, India; 2 Department of Pathology, All India Institute of Medical Sciences, Rajkot, Gujarat, India

**Keywords:** Malaria, peripheral blood smear microscopy, immunochromatographic rapid diagnostic card test, diagnostic performance

## Abstract

Malaria remains a major public health concern and accurate diagnosis is essential for effective management. This retrospective study compared the diagnostic performance of immunochromatographic rapid diagnostic tests (RDT) and peripheral blood smear (PBS) microscopy in India. A total of 150 suspected malaria cases were tested using both methods, with PBS confirming 30 positives (20%) and RDT detecting 20 positives (13.33%), all as *Plasmodium vivax*. PBS detected both *P. vivax* and *P. falciparum*, while RDT failed to identify *P. falciparum* but detected two *P. vivax* cases missed by PBS. Thus, we show that while RDT cannot fully replace PBS microscopy, it serves as a useful complementary tool for malaria diagnosis.

## Background:

Malaria is widespread in tropical areas, with around 247 million cases reported globally in 2021 across 84 countries where the disease is endemic [1]. In India, malaria poses a significant public health challenge, accounting for 1.7% of global malaria cases and 1.2% of malaria-related deaths, which includes 52% of all malaria fatalities outside of sub-Saharan Africa. Additionally, India carries 85.2% of the malaria burden in Southeast Asia and is responsible for 47% of the global incidence of *Plasmodium vivax* malaria, highlighting its crucial role in the global effort to eliminate malaria, especially in Southeast Asia. Malaria remains a major health threat in India, ranking among the top causes of morbidity and mortality from infectious diseases [2]. A significant challenge in managing malaria is the insufficient availability of dependable diagnostic and therapeutic solutions [3]. The early signs of malaria, including fever with chills, rigors, nausea, vomiting, headaches, muscle pain, fatigue and abdominal pain, are nonspecific and may differ, complicating clinical diagnosis. Prompt treatment is vital to prevent complications. The vague characteristics of malaria symptoms can result in both over-treatment and missed diagnoses, particularly in areas with low transmission rates. Therefore, precise diagnosis and species identification are critical [4]. Different diagnostic methods for malaria vary in terms of sensitivity, specificity, predictive values, efficiency, cost-effectiveness and user-friendliness [5]. Although the traditional examination of peripheral blood smears (PBS) is regarded as the gold standard for malaria diagnosis, it is labor-intensive and requires skilled professionals. New diagnostic techniques have been developed to possibly replace conventional microscopic methods. Therefore, it is of interest to evaluate the diagnostic utility of the Immunochromatographic Rapid Diagnostic Card Test (RDT) for malarial antigen and PBS microscopy in diagnosing malaria at a tertiary care centre.

## Material and Methods:

A retrospective record-based observational study was conducted in the Department of Microbiology and Pathology at a tertiary care health centre in Rajkot from January 2022 to December 2024. All blood samples that tested positive for malaria by any diagnostic method from 2022 to 2024 were included in the study. Malaria testing was conducted at the request of clinicians based on patient history and clinical examination. All samples received were subjected to an immunochromatographic test to detect Plasmodium species antigen (lactate dehydrogenase/aldolase) and *Plasmodium falciparum*-specific antigen (Histidine-rich protein 2), as well as PBS examination using thin and thick smears. Patient details, including name, age, sex, fever symptoms and clinical examination findings, were recorded. Sensitivity, specificity, positive predictive value (PPV) and negative predictive value (NPV) were calculated using standard formulas and a comparative analysis was conducted between PBS and RDT for all patients.

## Results:

Among the 150 blood samples analysed using PBS microscopy, 30 (20%) were found to be positive for malaria. Of these, 26 (86.66%) were identified as *Plasmodium vivax* and 4 (13.33%) as *Plasmodium falciparum*. When tested with RDT, 20 (13.33%) of the 150 samples were positive for malaria, all of which (100%) were *Plasmodium vivax*, with no cases of *Plasmodium falciparum* detected (Figure 1 - see PDF and 2 - see PDF). The diagnostic performance of the RDT is compared to PBS microscopy (Table 1 - see PDF, 2 - see PDF). The RDT's overall sensitivity and specificity for diagnosing malaria were 72% and 98%, respectively, with a PPV of 94% and an NPV of 90% (Table 3 - see PDF). Of the 30 malaria cases attributed to non-falciparum Plasmodium species (as diagnosed by PBS microscopy), RDT detected 20 cases (Figure 3A - see PDF), but missed 12 cases, resulting in false negatives. PBS identified all 4 cases of malaria caused by *Plasmodium falciparum* (Figure 3B - see PDF), while RDT failed to detect any, resulting in 4 false negatives. A total of 18 cases were positive by both RDT and PBS microscopy. Additionally, RDT alone diagnosed two cases of non-falciparum Plasmodium species, which were false positives.

## Discussion:

Malaria, a significant parasitic disease of global concern, poses a substantial public health challenge in India, leading to considerable morbidity, mortality and economic strain. The nation's strategy for addressing malaria emphasizes prompt diagnosis and immediate treatment to alleviate the related health consequences [6]. Despite the advancement of numerous diagnostic tests throughout the years, PBS microscopy continues to be regarded as the benchmark for assessing the effectiveness of these alternatives. While this technique is economical, it requires a significant degree of technical skill, stringent quality assurance and a regulated laboratory environment. Furthermore, it is labor-intensive, complex and requires considerable time to execute [7]. Recently, RDT has been recognized as a more sophisticated technique for identifying malarial antigens. This non-microscopic diagnostic method offers numerous benefits compared to conventional microscopy, such as quicker results, simpler implementation and easier interpretation. It necessitates minimal financial investment, functions without the need for electricity or additional tools and requires less technical expertise, contributing to its growing acceptance as a more effective diagnostic alternative for malaria [8]. Nonetheless, despite their benefits, these diagnostic techniques have certain drawbacks. They can be expensive and may yield inconsistent outcomes, as demonstrated by numerous studies. False positives may occur due to ongoing antigenemia or individuals taking antimalarial drugs on their own during fever episodes [9]. On the other hand, false negatives can arise from various factors, including cross-reactivity with autoantibodies like Rheumatoid Factor or heterophile antibodies, the development of immune complexes in severe cases of malaria and the prozone effect, among other causes [10]. In our ongoing research, we found that antigen card tests demonstrated a sensitivity of 72%, a specificity of 98.7%, a PPV of 94% and a NPV of 90% when evaluated against PBS microscopy. These findings are consistent with those reported in other studies [11, 12]. However, in this study, when comparing PBS with RDT, 18 cases were positive by both methods, while PBS failed to detect 02 cases that were positive by RDT, which is similar to findings in other studies [13]. The RDT shows significant promise as an effective tool for quick malaria detection, as demonstrated by our study's findings. The test's high positive diagnostic and low negative diagnostic likelihood ratios highlight its superior capability to accurately identify individuals with malaria compared to those without the disease. This indicates that the RDT could be crucial in enabling timely and accurate malaria diagnoses. Although this research does not suggest that the RDT for malarial antigens can completely replace PBS microscopy as a diagnostic method at this time, it implies that the rapid card test can serve as an alternative or supplementary tool to microscopy. This is particularly important in remote rural areas that frequently experience prolonged power outages and lack skilled technicians, laboratory facilities and other essential resources. Nonetheless, PBS microscopy will remain the gold standard when conducted by experts.

## Conclusion:

Malaria is often underdiagnosed when using conventional methods. Rapid diagnostic techniques should serve as complementary tools rather than replacements. RDT is quick, requires no specialized skills and is useful for routine diagnosis. However, the PBS method remains superior for accurate species differentiation, parasite quantification and maintaining permanent records. Thus, we show that RDT should be used alongside microscopy to enhance malaria diagnosis.

## Author contributions:

Mayuri Bhise and Garima Anandani were responsible for data collection, analysis, preparation and submission of the manuscript conceptualization and critical data analysis. Parth Goswami and Abhishek Padhi were responsible for the literature search and Ashwini Agarwal was accountable for reviewing and supervising the manuscript. All authors contributed to the article and approved the submitted version. Manuscript has been read and approved by all the authors and each author believes that the manuscript represents honest work.

## Funding:

No funds received or used

## Disclosure of ethical statements:

A retrospective record-based study approved by Institutional Ethics committee of AIIMS Rajkot: IEC Approval Number- IEC/AIIMS/RAJKOT/5th/ER/08. The authors confirm that we have adhered to the ethical policies of the journal. No identification details of the patients are shared.
